# The in vitro real-time oscillation monitoring system identifies potential entrainment factors for circadian clocks

**DOI:** 10.1186/1471-2199-7-5

**Published:** 2006-02-16

**Authors:** Yasukazu Nakahata, Makoto Akashi, Daniel Trcka, Akio Yasuda, Toru Takumi

**Affiliations:** 1 Osaka Bioscience Institute, Suita, Osaka 565-0874, Japan; 2 Life Science Laboratory, Material Laboratories, Sony Corporation, Shinagawa, Tokyo 144-0001, Japan

## Abstract

**Background:**

Circadian rhythms are endogenous, self-sustained oscillations with approximately 24-hr rhythmicity that are manifested in various physiological and metabolic processes. The circadian organization of these processes in mammals is governed by the master oscillator within the suprachiasmatic nuclei (SCN) of the hypothalamus. Recent findings revealed that circadian oscillators exist in most organs, tissues, and even in immortalized cells, and that the oscillators in peripheral tissues are likely to be coordinated by SCN, the master oscillator. Some candidates for endogenous entrainment factors have sporadically been reported, however, their details remain mainly obscure.

**Results:**

We developed the *in vitro *real-time oscillation monitoring system (IV-ROMS) by measuring the activity of luciferase coupled to the oscillatory gene promoter using photomultiplier tubes and applied this system to screen and identify factors able to influence circadian rhythmicity. Using this IV-ROMS as the primary screening of entrainment factors for circadian clocks, we identified 12 candidates as the potential entrainment factor in a total of 299 peptides and bioactive lipids. Among them, four candidates (endothelin-1, all-trans retinoic acid, 9-cis retinoic acid, and 13-cis retinoic acid) have already been reported as the entrainment factors *in vivo *and *in vitro*. We demonstrated that one of the novel candidates, 15-deoxy-Δ^12,14^-prostaglandin J_2 _(15d-PGJ_2_), a natural ligand of the peroxisome proliferator-activated receptor-γ (PPAR-γ), triggers the rhythmic expression of endogenous clock genes in NIH3T3 cells. Furthermore, we showed that 15d-PGJ_2 _transiently induces *Cry1*, *Cry2*, and *Rorα *mRNA expressions and that 15d-PGJ_2_-induced entrainment signaling pathway is PPAR-γ – and MAPKs (ERK, JNK, p38MAPK)-independent.

**Conclusion:**

Here, we identified 15d-PGJ_2 _as an entrainment factor *in vitro*. Using our developed IV-ROMS to screen 299 compounds, we found eight novel and four known molecules to be potential entrainment factors for circadian clocks, indicating that this assay system is a powerful and useful tool in initial screenings.

## Background

Circadian rhythms are endogenous self-sustained oscillations with approximately 24-hr rhythmicity that are manifested in various physiological and metabolic processes [1,2] In mammals the circadian orchestration of these processes is governed by pacemaker cells located within the suprachiasmatic nuclei (SCN) of the hypothalamus. It has also revealed that in mammals circadian oscillators exist not only in the SCN but also in peripheral tissues, and even in immortalized cells [3-5]. Because the periodicity of the circadian clock only approximates that of the environment, circadian clocks have to be adjusted to 24-h/day period by environmental time cues [6]. Circadian clocks are primarily synchronized with environmental time by the daylight cycle as an input signal to the SCN through the direct and indirect neural projections from retinal ganglion cells [6,7], however, other non-photic cues can also synchronize circadian clocks to 24-h/day [2].

The molecular mechanism of the circadian oscillator as a transcriptional-translational feedback loop has been unraveled by genetic analysis in *Drosophila *and mammals [8,9]. These molecular mechanisms based on the transcriptional-translational regulation are conserved among many species, including *Arabidopsis*, *Neurospora*, *Drosophila*, zebrafish, and mammals [9-11]. In mammals, principally two basic helix-loop-helix-PAS transcriptional factors, CLOCK and BMAL1, regulate gene expression by interacting with a promoter element termed E-box [12,13]. Target genes of these transcriptional factors include several repressor proteins, including PER1, PER2, PER3, CRY1, and CRY2, which function to inhibit the activity of CLOCK/BMAL1 complex by entering into the nucleus [14,15], thereby generating a circadian oscillation of their own transcription.

One of the molecular features of circadian clocks is rhythmic fluctuation of clock gene mRNA amounts. *In situ *hybridization and RNase protection assay are conventional techniques used to detect expression profiles of the clock and clock-controlled genes (for example, [4,16,17]). Quantitative real-time RT-PCR has recently become a popular method to investigate mRNA expression profiles (for example, [5,18]). The bioluminescent firefly luciferase protein has proven to be a useful reporter protein for monitoring the dynamics of gene activity in living cells [19]. Luminescence from luciferase expressed in transgenic plants, *Drosophila*, zebrafish and mammals has been used to monitor real-time dynamic change in gene transcription within the living organism [19-22]. Since this system is applied to transiently transfected cell cultures with clock gene promoters driving firefly luciferase gene expression [5,23], luciferase real-time monitoring system using photomultiplier tubes has become a powerful tool to investigate circadian clock mechanism, in particular to identify the critical elements for producing the circadian rhythmicity [24-26].

As described above, it has been thought that circadian clocks in peripheral tissues are regulated by the SCN via the secretion of hormones and/or the sympathetic/parasympathetic innervations from the SCN to peripheral tissues [27]. Recently, some potential "entrainment factors" have been reported [28-32], however, the mechanisms how the central SCN pacemaker clock orchestrates the peripheral clocks remains unclear. Here, we report systematic screening of various molecules in attempt to find entrainment factors by using our *in vitro *real-time oscillation monitoring system (IV-ROMS). In this study, we report eight novel candidates, including 15-deoxy-Δ^12,14^-prostaglandin J_2 _(15d-PGJ_2_), of entrainment factors for circadian clocks.

## Results and discussion

### Establishment of IV-ROMS using mPer2-luc/Rat1 cell lines

**Figure 1:**
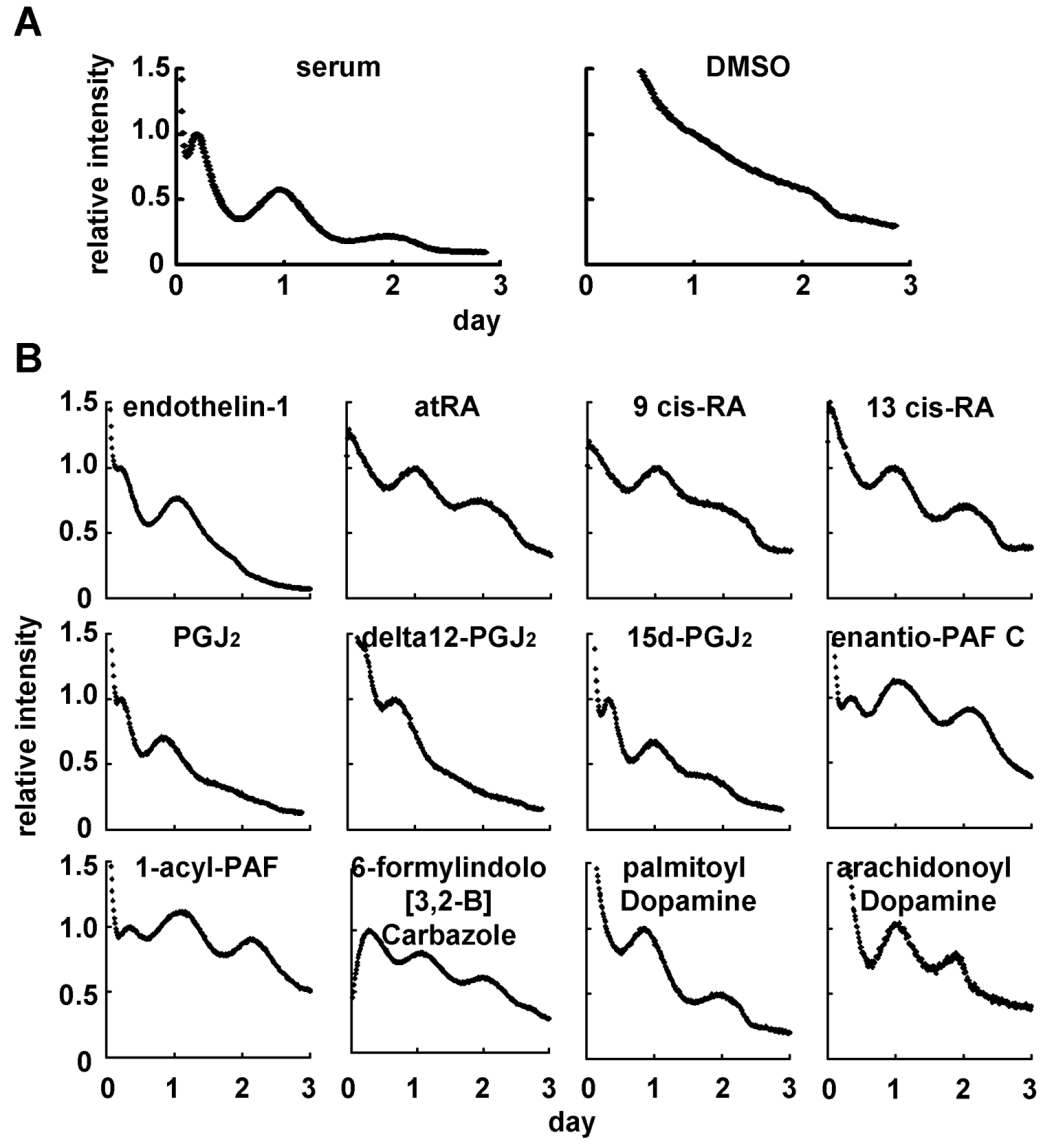
**Screening of entrainment factors by using IV-ROMS**. *mPer2*-luc/Rat1 cells were monitored the luciferase intensity for duration of 3 days using IV-ROMS. Figure 1A shows luciferase activity after 1 h treatment with 50% serum (positive control, left panel) or DMSO (negative control, right panel). Figure 1B shows luciferase activity after 1 h treatment with 1 or 10 μM of each of the 12 possible circadian entrainment factors. A representative result was chosen out of three independent experiments. Abscissa presents "day", ordinate "relative luciferase intensity", respectively. The first peak was set to 1.

The photomultiplier tube detector system can detect bioluminescence of luciferase protein and can measure it every 15 min using a turntable (see Additional file 1). This system is maintained in an incubator at 5% CO_2 _and 35°C. We first established *mPer2*-luc/Rat1 fibroblast cell lines that stably express luciferase gene driven by *mPer2 *promoter. *Per2 *is considered to be one of the core molecule for molecular clocks since gene-knockout analysis revealed that *mPer2 *mutants display a shorted circadian period followed by a loss of circadian rhythmicity in constant darkness [33]. After stimulation with high concentration of serum for 1 h, rhythmicity of luciferase activity was monitored for duration of at least 2 or 3 days (Fig. 1A). In contrast to no oscillation in control (by DMSO treatment), rhythmic activity of luciferase was observed. Rhythmic phase of *mPer2*-luc/Rat1 cell lines was phenotypically the same as that in transiently transfected cells with *mPer2*-luc construct and was antiphase compared to transient transfected cells with *hBmal1*-luc construct [5]. These cell lines showed no abnormalities in their cell growth and morphology. In summary, these cell lines established here are suitable for the screening assay designed to identify entrainment factors for circadian clocks.

### Screening of peptide and bioactive lipid libraries for circadian entrainment factors

The results of screening are shown in Figure 1B and Additional file 2 by using Peptide library (BAP96S, assayscript, Osaka, Japan) and Bioactive lipid library (Version 3, BIOMOL, PA, USA). Out of 299 compounds screened, 12 demonstrated the rhythmic expression of luciferase. Among them, four compounds (endothelin-1, all-trans retinoic acid, 9-cis retinoic acid, and 13-cis retinoic acid) have already been reported as resetting factors *in vivo *or *in vitro *[30,34]. By this assay, we newly identified eight candidates for circadian entrainment factors; prostaglandin J_2 _(PGJ_2_), Δ^12^-PGJ_2_, 15-deoxy-Δ^12,14^-PGJ_2 _(15d-PGJ_2_), enantio-PAF C16, 1-acyl-PAF, 6-formylindolo [3,2-B] carbazole, palmitoyl dopamine, and arachidonoyl dopamine. These two libraries contain five known entrainment factors and we could identify all of them, except prostaglandin E_2 _[32], as an entrainment factor by this assay system (see Additional file 2), indicating that this assay system is reliable and suitable for screening of entrainment factors. We could not identify prostaglandin E_2 _because prostaglandin E_2 _receptor EP1, which is responsible for the entrainment of circadian clocks, was not expressed in Rat1 cells, but was expressed in NIH3T3 cells that Tsuchiya et al used [32] (see Additional file 3).

### 15d-PGJ_2 _triggers the rhythmic expression of endogenous clock genes in NIH3T3 cells

**Figure 2:**
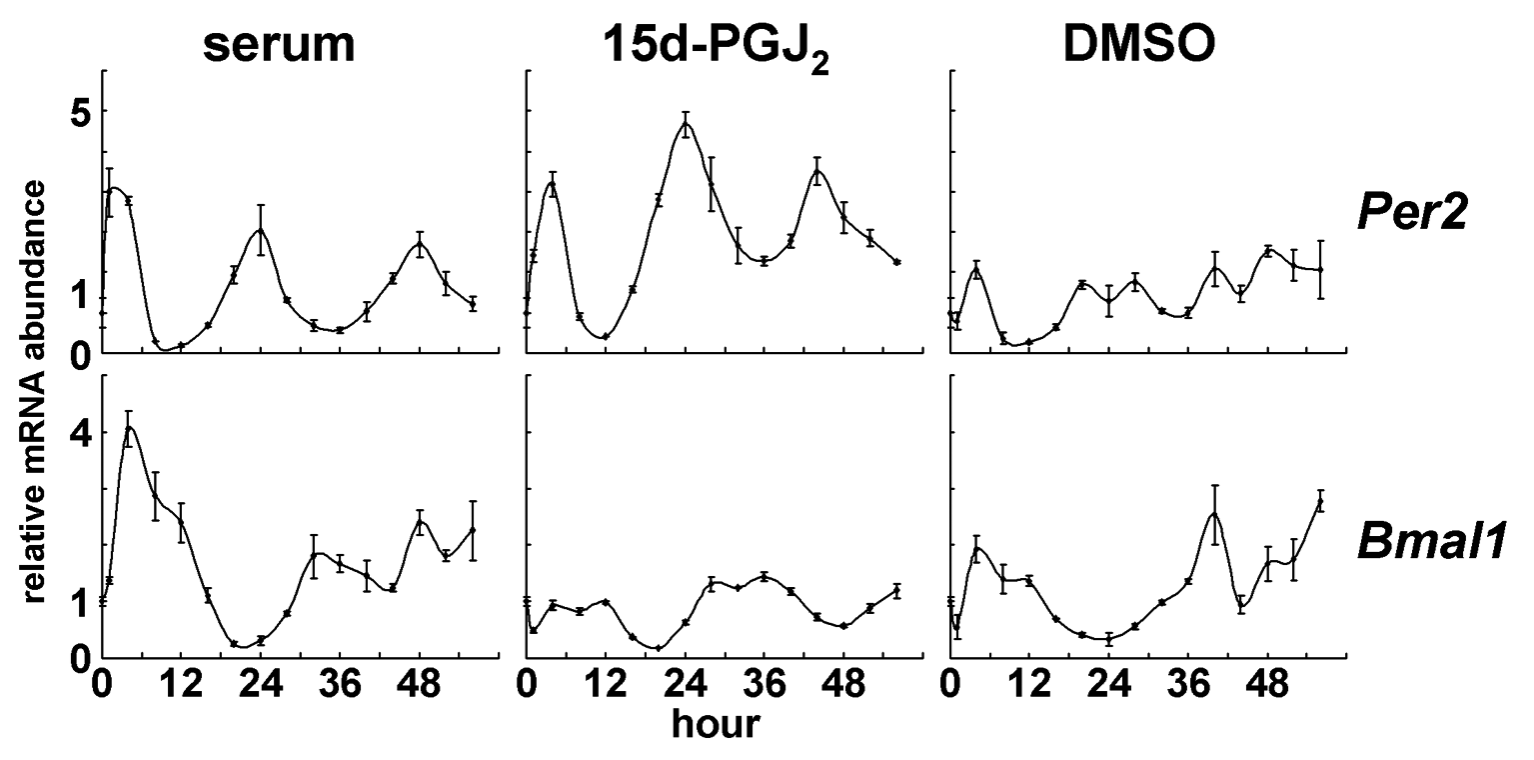
**Endogenous clock gene expression profiles stimulated by 15d-PGJ_2_**. NIT3T3 cells were stimulated by 50% serum (positive control; left column), DMSO (negative control; right column), or 10 μM 15d-PGJ_2 _(middle panel) for 1 h. Total RNAs were isolated at each time point. Quantitative real-time RT-PCR was performed using *mPer2 *(upper panels), *mBmal1 *(bottom panels), and 18S rRNA primers. Abscissa presents "hour", ordinate "mRNA amount", respectively. mRNA amount at time 0 was set to 1. The relative levels of each mRNA were normalized to the corresponding 18S rRNA levels.

Among the eight novel candidates for entrainment factors, we focused on 15d-PGJ_2_, because cells stimulated by 15d-PGJ_2 _displayed the most robust effects on rhythmicity. 15d-PGJ_2 _has recently received increasing attention because it functions as a potential regulator of diverse processes including cell growth, proliferation, differentiation, and inflammation [35]. In addition, 15d-PGJ_2 _is the dehydration end product of PGD_2_. Interestingly, PGD_2 _has been recognized as the most potent endogenous sleep-promoting substance [36]. Furthermore, the PGD_2 _concentration in rat cerebrospinal fluid shows a circadian shift coupled to the sleep-wake cycle [37]. To confirm whether 15d-PGJ_2 _is an authentic endogenous entrainment factor, we examined the expression profiles of clock genes in NIH3T3 fibroblast cells stimulated by 15d-PGJ_2_. *Per2 *and *Bmal1 *expression patterns were examined by quantitative real-time RT-PCR at 4-h intervals for duration of 56 h and rhythmic expressions were observed when treated for 1 h with 15d-PGJ_2 _and with high concentration serum, but not when treated with DMSO as a control (Fig. 2). Moreover, phases of *Per2 *and *Bmal1 *mRNA expression triggered by 15d-PGJ_2 _treatment were antiphasic with respect to each other, which is consistent with those triggered by serum and with previously reported expression profiles [5,38]. Taken together, these results demonstrate that 15d-PGJ_2 _can act as an *in vitro *entrainment factor for circadian clocks. PGD_2 _and other prostaglandins and prostanoids examined in this study showed no rhythmic fluctuation of luciferase activity (data not shown). The facts that 15d-PGJ_2_'s precursor PGD_2 _has been recognized as the most potent endogenous sleep-promoting substance and that the PGD_2 _concentration in rat cerebrospinal fluid shows a circadian change coupled to the sleep-wake cycle, have led to the hypothesis that 15d-PGJ_2 _may act as an endogenous circadian entrainment factor *in vivo*. It would be interesting to see the effects of 15d-PGJ_2 _*in vivo*. However, it should be noted that the endogenous concentration of 15d-PGJ_2 _is extremely low [39], compared with the one used for the *in vitro *screening.

### 15d-PGJ_2 _up-regulates Cry1, Cry2, and Rorα mRNA expressions

**Figure 3:**
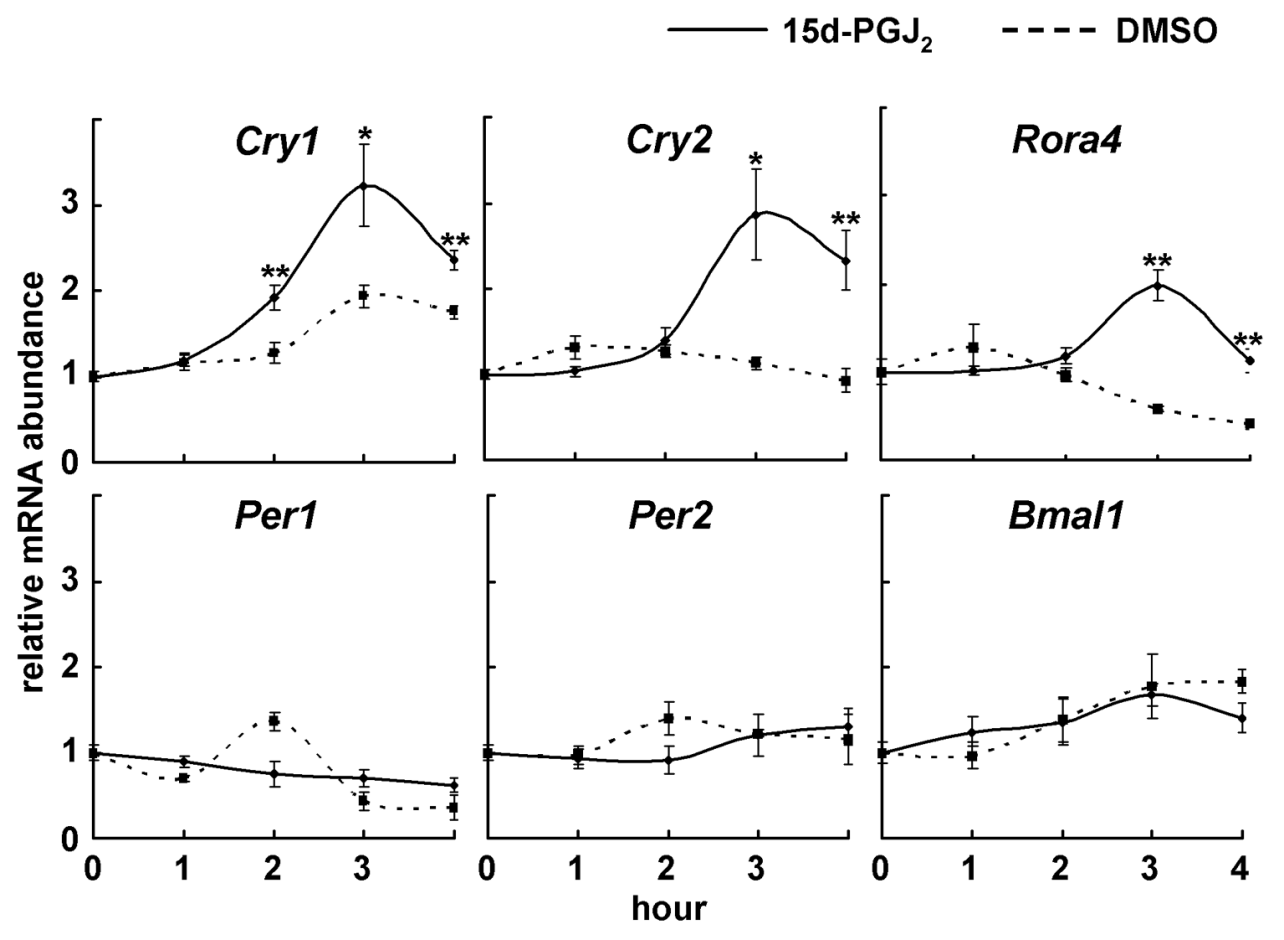
**15d-PGJ_2 _up-regulates *Cry1*, *Cry2*, and *Rorα *mRNA expression**. NIH3T3 cells were stimulated by 10 μM 15d-PGJ_2 _(solid line) or DMSO (dotted line) for 1 h. Total RNAs were isolated at each time point. Quantitative real-time RT-PCR was performed using *mCry1*, *mCry2*, *mRorα*, *mPer1*, *mPer2*, *mBmal1*, and 18S rRNA primers. Abscissa presents "hour", ordinate "mRNA amount", respectively. Each mRNA amount at time point 0 was set to 1. Data are shown as the mean ± SE from three to five independent experiments. The relative levels of each mRNA were normalized to the corresponding 18S rRNA levels. *p < 0.05, **p < 0.01 compared with relative control (Student's t-test).

To examine which clock genes are induced by 15d-PGJ_2 _treatment, we systematically quantified the expression levels of the canonical clock genes. After the isolation of total RNA at 1-h intervals from NIH3T3 cells stimulated by 15d-PGJ_2_, quantitative real-time RT-PCR was performed using primers for *Per1*, *Per2*, *Per3*, *Bmal1*, *Npas2*, *Cry1*, *Cry2*, *Dec1*, *Dec2*, *E4bp4*, *Dbp*, and *Rorα *by using low-density arrays. Unexpectedly, stimulation with 15d-PGJ_2 _did not affect a transient *Per1 *and *Per2 *mRNA accumulation (Fig. 3), although both *Per *genes are known to be transiently accumulated by the various stimuli of entrainment [40]. On the other hand, we for the first time found that 15d-PGJ_2 _induced accumulation of *Cry1 *and *Cry2 *transcripts, as well as *Rorα *mRNA (Fig. 3), which is consistent with a previous report [41].

### Entrainment triggered by 15d-PGJ_2 _is independent of PPAR-γ signaling pathway

**Figure 4:**
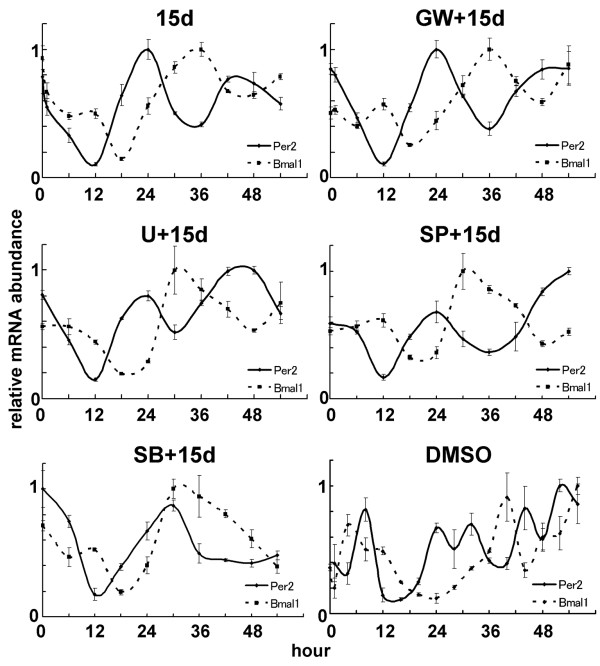
**Rhythmic clock gene expression triggered by 15d-PGJ_2 _is independent of PPAR-γ-, MAPK-, JNK- and p38MAPK-signaling pathway**. After 1 h pretreatment of DMSO (15d; upper left panel), 10 μM GW9662 (GW+15d; upper right panel), 10 μM U0126 (U+15d; middle left panel), 20 μM SP600125 (SP+15d; middle right panel), or 30 μM SB203580 (SB+15d; bottom left panel), NIH3T3 cells were stimulated by 10 μM 15d-PGJ_2 _for 1 h. As a negative control, NIH3T3 cells were pretreated by DMSO and stimulated by DMSO for 1 h (DMSO; bottom right panel). Total RNAs were isolated at each time point. Quantitative real-time RT-PCR was performed using *mPer2 *(solid line), *mBmal1 *(dotted line), and 18S rRNA primers. Abscissa presents "hour", ordinate "mRNA amount", respectively. The maximum mRNA amount was set to 1. The relative levels of each mRNA were normalized to the corresponding 18S rRNA levels.

We next sought to identify entrainment signaling pathways triggered by 15d-PGJ_2_. Since 15d-PGJ_2 _has been known to be a natural ligand of the peroxisome proliferator-activated receptor-γ (PPAR-γ) [42], we assessed whether the clock gene expression triggered by 15d-PGJ_2 _is dependent on the PPAR-γ-mediated signaling pathway. NIH3T3 cells pretreated with DMSO or with a specific irreversible PPAR-γ antagonist GW9662 [43] were then stimulated with 15d-PGJ_2_, and harvested at every 6 h for duration of 54 h. Quantitative real-time RT-PCR using primers for *Per2 *and *Bmal1 *showed no different expression patterns between GW9662 and DMSO pretreated cells (Fig. 4). The same concentration (10 μM) of 15d-PGJ_2 _induced GADD45 and catalase mRNA, which are induced via PPAR-γ, in the same NIH3T3 cells, however, no stimulation of both mRNAs was seen in these cells pretreated with 10 μM GW9662 (see Additional file 4), indicating that these cells express PPAR-γ, that PPAR-γ was involved in our observation, and that the amount of GW9662 we used was enough for the system to work. These results suggest that the circadian entrainment triggered by 15d-PGJ_2 _is independent of the PPAR-γ signaling pathway. We further confirmed that other PPAR-γ ligands, Ciglitazone [44] and hexadecyl azelaoyl phosphatidlycholine (azPC) [45], did not lead to the circadian expression of the clock genes (see Additional file 5).

We then explored which signaling pathways are involved in 15d-PGJ_2_-induced rhythmic clock gene expression. Recently, administration of 15d-PGJ_2 _was shown to activate ERK and JNK signaling pathways [42,46]. The known entrainment factors are thought to mainly act by activating ERK signaling pathway [47]. We thus examined whether these two MAPK signaling pathways can be linked with 15d-PGJ_2_-induced cyclic gene expression. Surprisingly, pretreatment of a specific JNK inhibitor SP600125 [48] and of a specific MEK inhibitor U0126 [49], both showed no effect on the entrainment of circadian clocks (Fig. 4). Although results of MAPK/ERK to entrainment in the different systems have been inconsistent [50], these results suggest that there exists an unknown entrainment pathway, independent of the ERK-mediated signaling pathway. Meanwhile, another pathway, the p38 MAPK signaling pathway was recently shown to be associated with circadian clocks by modulating their period lengths [51]. SB203580, a specific p38 inhibitor [52], slightly delayed the phase of *Per2 *rhythms but did not affect circadian expression of both *Per2 *and *Bmal1 *(Fig. 4), indicating that p38 MAPK signaling pathway is involved in modulation of period length, but not in the induction of clock gene expression by 15d-PGJ_2_.

**Figure 5:**
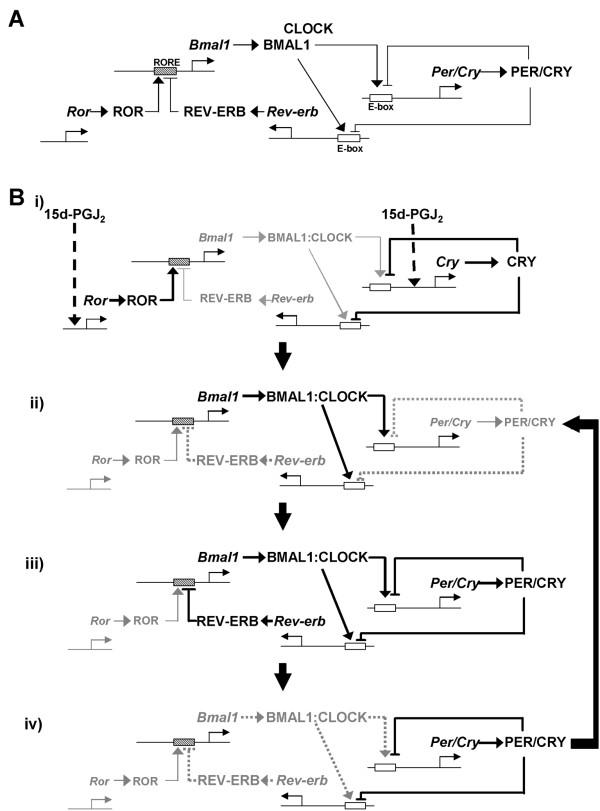
**Schema of the entrainment mechanism by 15d-PGJ_2_**. 15d-PGJ_2 _up-regulates transcription of *Cry*s and *Rorα *(Fig. 5Bi). The translated RORα activates *Bmal1 *transcription, and translated BMAL1 then binds to CLOCK and their heterodimers activate *Per*/*Cry *and *Rev-erb *transcriptions via E/E' box (Fig. 5Bii). Translated PER/CRY and REV-ERB inhibit *Per*/*Cry*/*Rev-erb *and *Bmal1 *transcription, respectively (Fig. 5Biii). The inhibition of *Rev-erb *transcription reduces *Bmal1 *transcription (Fig. 5Biv). The reduced *Per*/*Cry *transcription and relatively increased RORα activity (by inhibition of REV-ERB) again up-regulate *Bmal1 *transcription (Fig. 5Biv -> 5Bii).

The interpretation of this study on the transcription-translation feedback loops of clock genes are summarized in Figure 5A. As shown in Figure 3, 15d-PGJ_2 _up-regulates transcription of *Cry*s and *Rorα *(Fig. 5Bi). The translated RORα activates *Bmal1 *transcription [24,53,54], and translated BMAL1 binds to CLOCK forming a heterodimer which activates *Per*/*Cry *and *Rev-erb *transcriptions via E/E' elements (Fig. 5Bii; [12,13]). Translated PER/CRY and REV-ERB inhibit transcription of *Per*/*Cry*/*Rev-erb *and *Bmal1 *genes, respectively (Fig. 5Biii). The inhibition of *Rev-erb *transcription also reduces *Bmal1 *transcription (Fig. 5Biv; [55]). The reduced *Per*/*Cry *transcription and relatively increased RORα activity (by inhibition of REV-ERB) again up-regulate *Bmal1 *transcription and result in a completion of the "loop" (Fig. 5Biv -> 5Bii).

### In vitro real-time oscillation monitoring system (IV-ROMS)

IV-ROMS can be applied to identify molecules which are involved in other mechanisms pertaining to circadian clock system; transcriptional-translational feedback loops of circadian mechanism and input signaling pathway mechanism, for example, by using RNAi, inhibitor, and other libraries. We can also apply this system to other research fields. Further modification and development of this system will be needed in order to be applied for more systematic and high-throughput screenings.

## Conclusion

Here we present the *in vitro *real-time oscillation monitoring system (IV-ROMS). Indeed, we newly found eight candidates out of 299 compounds as circadian entrainment factors (Fig. 1 and Additional file 2). We further confirmed that one of the candidates, 15d-PGJ_2_, triggers the rhythmic expression of endogenous circadian clocks by inducing *Cry*s and *Rorα*, but not *Per*s, in NIH3T3 cells (Fig. 2), indicating that this assay system is a powerful and useful tool for the initial screening procedure. This system can also be applied not only to find new intracellular molecules involved in circadian clocks; new transcription factors, new signaling and degradation pathways, but also to investigate other cellular mechanisms like cell-cycle or oncogenesis.

## Methods

### Cell culture

Rat1 and NIH3T3 fibroblast cells were grown at 37°C and 5% CO_2_. Rat1 and NIH3T3 cells were grown in Dulbecco's Modified Eagle Medium (1.0 g/L glucose) with *L*-Gln and sodium pyruvate (DMEM, Nacalai tesque, Kyoto, Japan) supplemented with 5 and 10% fetal bovine serum (FBS), respectively, and antibiotics.

### Establishment of mPer2-luciferase-stably-expressing Rat1 cell line

A bacterial artificial chromosome (BAC) clone (pBeloBac11 24484) containing the complete genomic sequence of the mouse *Per2 *(*mPer2*) gene was purchased from BACPAC Resource Center at Children's Hospital Oakland Research Institute. The *mPer2 *promoter region was isolated and cloned in the pGL3 Basic vector (Promega). The *mPer2 *region spans from -2811 to +110 (+1 indicates the putative transcriptional start site). Rat1 cells were cotransfected with linearized *mPer2 *promoter/pGL3 and pcDNA3, which contains neomycin resistant gene. Transfection was carried out by using Polyfect Transfection Reagent (QIAGEN) according to the manufacture's instructions. The cells were cultured in 10% FBS/DMEM containing 500 μg/ml geneticin (SIGMA) for 1 to 2 weeks. Cells were then individually isolated, and 24 clones were established as *mPer2*-luc/Rat1 cells. After screening for the luciferase activity by using IV-ROMS, we established two independent clones with clear rhythmic activity.

### Real-time luciferase activity monitoring in living cells

*mPer2*-luc/Rat1 cells were seeded in a 35 mm-dish at density of 2 × 10^5 ^cells and incubated for 2 days. The medium was then exchanged for serum-free medium supplemented with a compound to be screened. Compound was diluted to a final concentration of 1 μM for peptide and 1 or 10 μM for bioactive lipid, respectively. One hour later the medium was replaced with 1% FBS/DMEM supplemented with 0.1 mM luciferin/10 mM HEPES (pH 7.2). Light emission was measured and integrated for 1 min at intervals of 15 min with a photomultiplier tube (Hamamatsu Photonics, Hamamatsu, Japan). Data were analyzed by LM2400 software (Hamamatsu Photonics).

### Real-time quantitative RT-PCR

TaqMan^® ^Low Density Array (Applied Biosystems), which contained *mPer1*, *mPer2*, *mPer3*, *mArntl *(*mBmal1*), *mNpas2*, *mCry1*, *mCry2*, *mBhlhb2 *(*mDec1*), *mBhlhb3 *(*mDec2*), *mDbp*, and *mNfil3 *(*mE4bp4*) as clock genes and 18S rRNA as an internal control, was examined by using an ABI PRISM 7900HT Sequence Detection System (Applied Biosystems) as described previously [56]. For one port of the TaqMan Low Density Array, 100 ng cDNA template was mixed with 50 μl of 2 × TaqMan Universal PCR Master Mix (Applied Biosystems) and filled up to 100 μl with distilled water. The reaction was first incubated at 50°C for 2 min, then at 95°C for 10 min, followed by 40 cycles of 95°C for 15 sec and 60°C for 1 min.

Each real-time quantitative RT-PCR was performed by using an ABI PRISM 7900HT Sequence Detection System as described previously [5]. Briefly, DNase-treated total RNA (1 μg) was reverse-transcribed by using a random hexamer primer and Superscript reverse transcriptase (Invitrogen). The cDNA equivalent to 50 ng total RNA was PCR-amplified. The PCR primers and TaqMan probes (5' FAM and 3' TAMRA) used are as follows:

*mPer2 *FW: CGC CTA GAA TCC CTC CTG AGA,

*mPer2 *RV: CCA CCG GCC TGT AGG ATC T,

*mPer2 *TaqMan probe: AGG CTG TGG ATG AAA GGG CGG TC,

*mBmal1 *FW: GCA GTG CCA CTG ACT ACC AAG A,

*mBmal1 *RV: TCC TGG ACA TTG CAT TGC AT,

*mBmal1 *TaqMan probe: ATC AAG AAT GCA AGG GAG GCC CAC A,

18S rRNA FW: CGC CGC TAG AGG TGA AAT TC,

18S rRNA RV: CGA ACC TCC GAC TTT CGT TCT,

18S rRNA TaqMan probe: CCG GCG CAA GAC GGA CCA GA.

The PCR primers for *mCry1*, *mCry2*, and *mRorα *were described previously [5,24]. Values are reported as mean ± SE. Statistical differences were determined by a Student's *t *test. Statistical significance is displayed as * (p < 0.05) or ** (p < 0.01).

## List of abbreviations used

SCN, suprachiasmatic nuclei; IV-ROMS, in vitro real-time oscillation monitoring system; 15d-PGJ_2_, 15-deoxy-Δ^12,14^-prostaglandin J_2_; PPAR-γ, peroxisome proliferators-activated receptor-γ; RT-PCR, reverse transcription-polymerase chain reaction; DMEM; Dulbecco's modified eagle medium; FBS, Fetal bovine serum

## Authors' contributions

YN carried out most of experiments and drafted the manuscript. MA designed the system. DT carried out the molecular biology studies and proofread the manuscript. AY participated in the coordination and provided financial support. TT is the PI and has participated in its design and coordination, and drafted the manuscript. All authors read and approved the final manuscript.

## Supplementary Material

Additional File 1 **Schema of IV-ROMS**. Two photomultiplier tubes are installed within the lid and the light emission is integrated for 1 min per sample at intervals of 15 min. 24 samples are able to be detected at once. Data are analyzed by LM2400 software (Hamamatsu Photonics). Click here for file

Additional File 2 **Compound libraries used in this assay**. Peptide library (BAP96S, assayscript, Osaka, Japan) and Bioactive lipid library (Version 3, BIOMOL, Plymouth Meeting, PA, USA) were used in this screening assay. Numbers in parentheses indicate the number of known entrainment factors. Click here for file

Additional File 3 **EP1 mRNA is expressed in NIH3T3 cells, but not in Rat1 cells**. Total RNAs were isolated from NIH3T3 and Rat1 cells and semi-quantitative real-time RT-PCR was performed using prostaglandin E2 receptor EP1 and G3PDH primers. G3PDH mRNA was used as an internal control. For EP1 and G3PDH, the cDNA equivalent to 50 ng and 0.5 ng of total RNA respectively, were PCR-amplified by 30 cycles each. Click here for file

Additional File 4 **Pretreatment of PPAR-γ antagonist, GW9662, repressed 15d-PGJ_2_-induced PPAR-γ-mediated gene expressions**. The effect of GW9662 on PPARγ-mediated GADD45 (A) and catalase (B) mRNA expressions was evaluated by using a quantitative real-time PCR. 1 h GW9662 (GW: +) or DMSO (GW: -) pretreated-NH3T3 cells were stimulated by 15d-PGJ_2 _(15d: +) or DMSO (15d: -). mRNA amounts after 1 h stimulation by15d-PGJ_2 _or DMSO were measured. The mRNA amount of GW: -, 15d: - condition was set to 1. The relative levels of each mRNA were normalized to the corresponding 18S rRNA levels. Data are shown as the mean ± SE from three independent experiments. *p < 0.05, **p < 0.01 compared with the condition 'GW: -, 15d: +'. (Student's t-test). Click here for file

Additional File 5 **15d-PGJ_2_-induced entrainment mechanism for circadian clocks is independent of PPAR-γ-signaling pathway**. After 1 h pretreatment with (middle right panel) or without (middle left panel) PPAR-γ antagonist, GW96662, *mPer2*-luc/Rat1 cells were stimulated by 15d-PGJ_2 _for 1 h and the luciferase intensity was monitored by using IV-ROMS. *mPer2*-luc/Rat1 cells stimulated for 1 h by PPAR-γ agonists, azPC (bottom left panel) or ciglitazone (bottom right panel) were monitored by using IV-ROMS. A representative result was chosen out of at least three independent experiments. Abscissa presents "day", ordinate "relative luciferase intensity", respectively. Click here for file
