# Supplementary figures and images for: The in vitro real-time oscillation monitoring system identifies potential entrainment factors for circadian clocks
Source: BMC Mol Biol. 2006 Feb 16;7:5. doi: 10.1186/1471-2199-7-5 (PMC1386696; doi:10.1186/1471-2199-7-5)

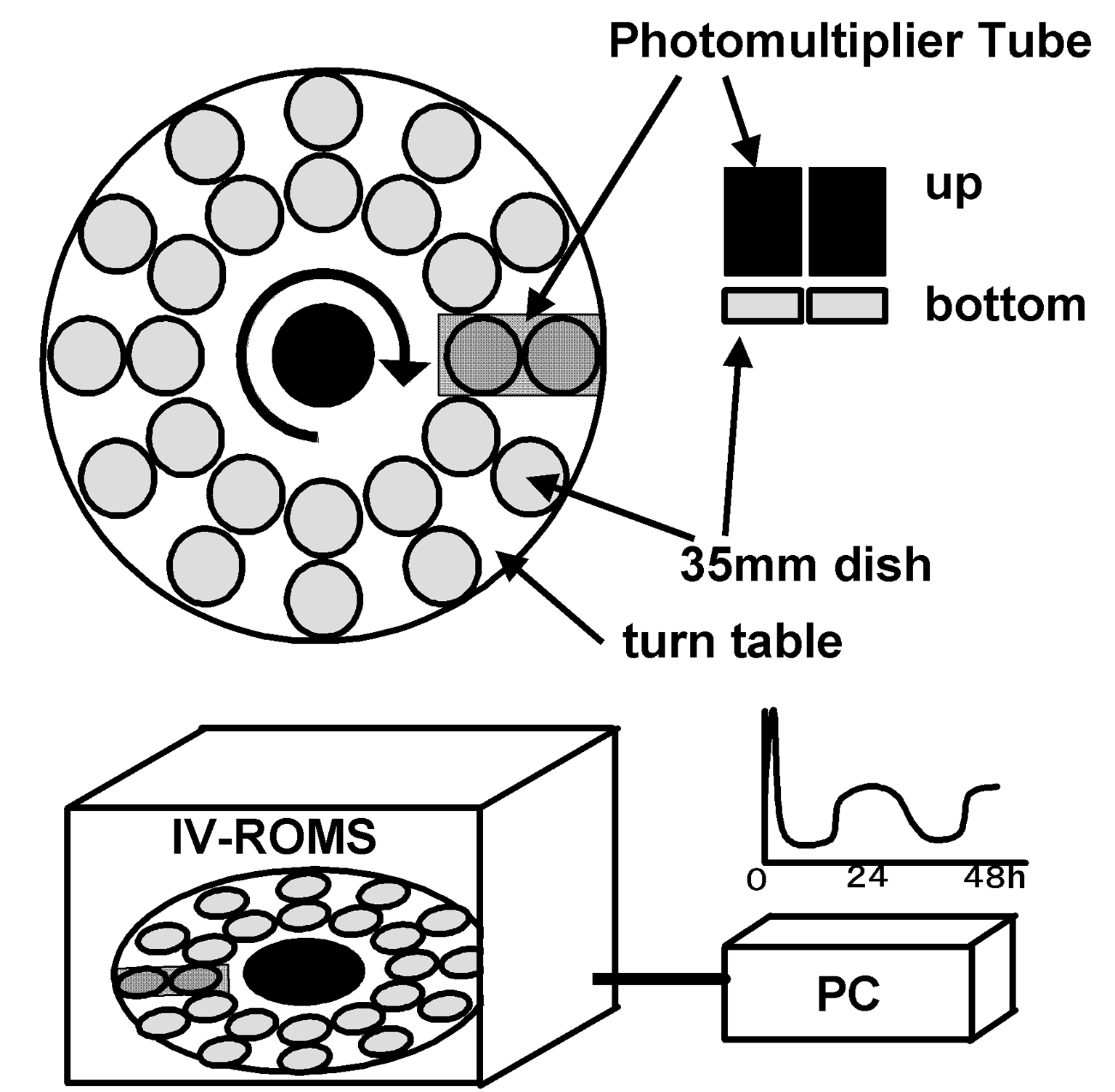

Supplement: Additional File 1 — Schema of IV-ROMS. Two photomultiplier tubes are installed within the lid and the light emission is integrated for 1 min per sample at intervals of 15 min. 24 samples are able to be detected at once. Data are analyzed by LM2400 software (Hamamatsu Photonics). [file 1471-2199-7-5-S1.png]

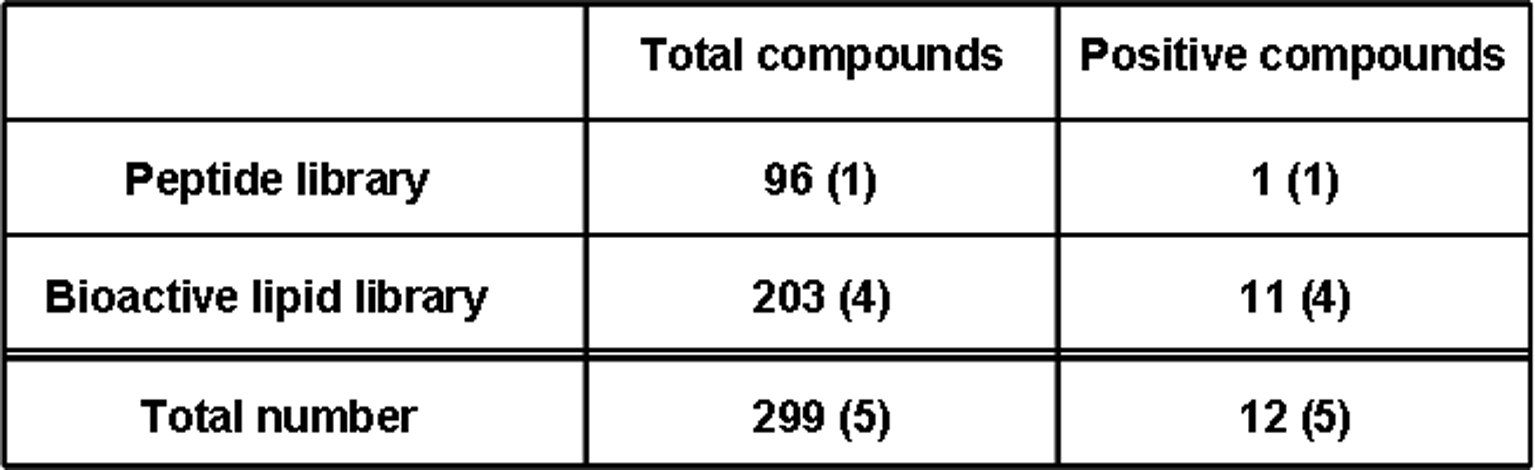

Supplement: Additional File 2 — Compound libraries used in this assay. Peptide library (BAP96S, assayscript, Osaka, Japan) and Bioactive lipid library (Version 3, BIOMOL, Plymouth Meeting, PA, USA) were used in this screening assay. Numbers in parentheses indicate the number of known entrainment factors. [file 1471-2199-7-5-S2.png]

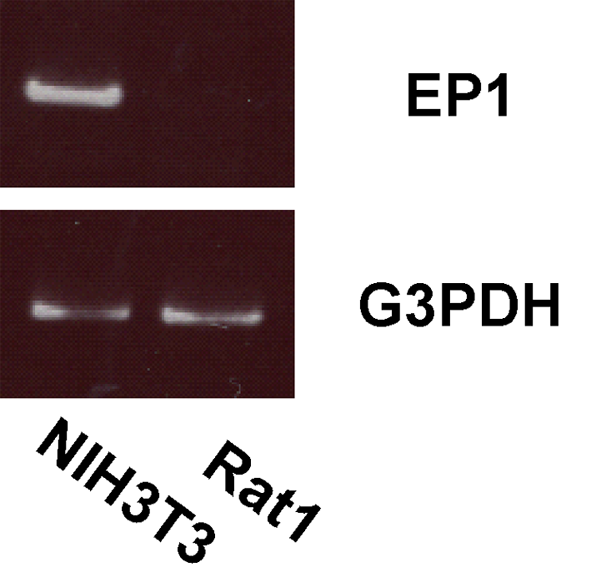

Supplement: Additional File 3 — EP1 mRNA is expressed in NIH3T3 cells, but not in Rat1 cells. Total RNAs were isolated from NIH3T3 and Rat1 cells and semi-quantitative real-time RT-PCR was performed using prostaglandin E2 receptor EP1 and G3PDH primers. G3PDH mRNA was used as an internal control. For EP1 and G3PDH, the cDNA equivalent to 50 ng and 0.5 ng of total RNA respectively, were PCR-amplified by 30 cycles each. [file 1471-2199-7-5-S3.png]

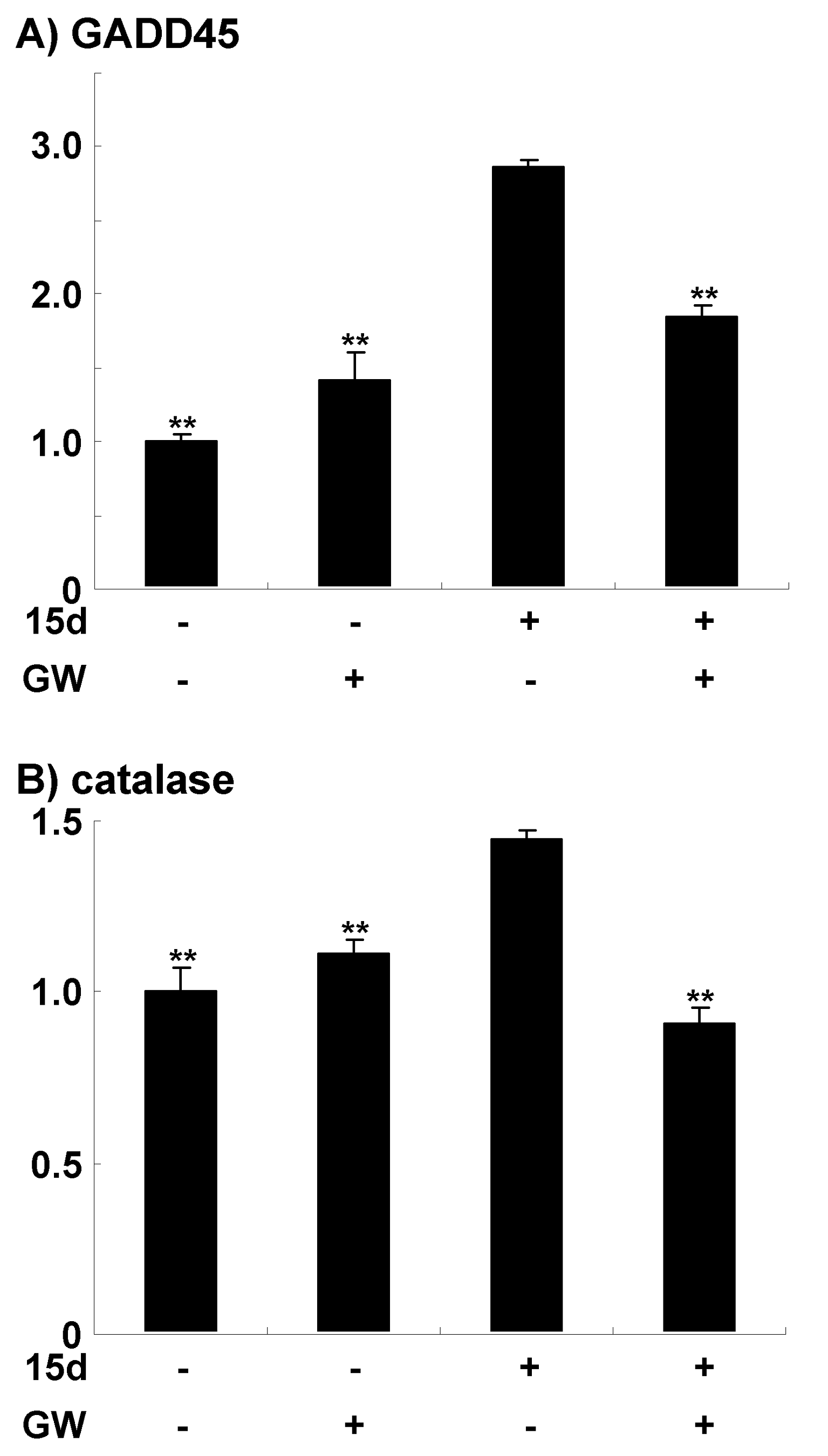

Supplement: Additional File 4 — Pretreatment of PPAR-γ antagonist, GW9662, repressed 15d-PGJ2-induced PPAR-γ-mediated gene expressions. The effect of GW9662 on PPARγ-mediated GADD45 (A) and catalase (B) mRNA expressions was evaluated by using a quantitative real-time PCR. 1 h GW9662 (GW: +) or DMSO (GW: -) pretreated-NH3T3 cells were stimulated by 15d-PGJ2 (15d: +) or DMSO (15d: -). mRNA amounts after 1 h stimulation by15d-PGJ2 or DMSO were measured. The mRNA amount of GW: -, 15d: - condition was set to 1. The relative levels of each mRNA were normalized to the corresponding 18S rRNA levels. Data are shown as the mean ± SE from three independent experiments. *p < 0.05, **p < 0.01 compared with the condition 'GW: -, 15d: +'. (Student's t-test). [file 1471-2199-7-5-S4.png]

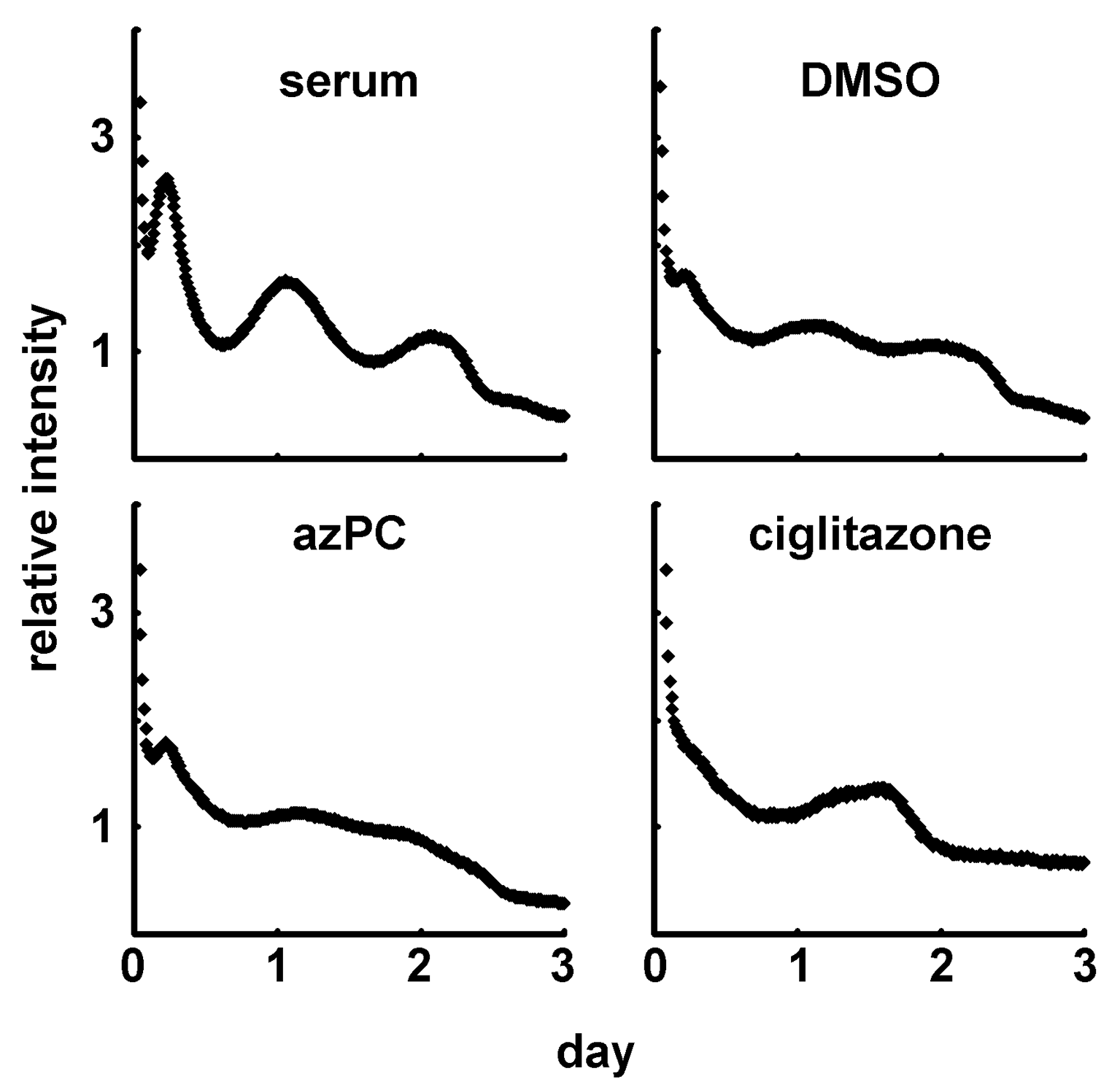

Supplement: Additional File 5 — 15d-PGJ2-induced entrainment mechanism for circadian clocks is independent of PPAR-γ-signaling pathway. After 1 h pretreatment with (middle right panel) or without (middle left panel) PPAR-γ antagonist, GW96662, mPer2-luc/Rat1 cells were stimulated by 15d-PGJ2 for 1 h and the luciferase intensity was monitored by using IV-ROMS. mPer2-luc/Rat1 cells stimulated for 1 h by PPAR-γ agonists, azPC (bottom left panel) or ciglitazone (bottom right panel) were monitored by using IV-ROMS. A representative result was chosen out of at least three independent experiments. Abscissa presents "day", ordinate "relative luciferase intensity", respectively. [file 1471-2199-7-5-S5.png]
